# Electrical activation of degenerated photoreceptors in blind mouse retina elicited network-mediated responses in different types of ganglion cells

**DOI:** 10.1038/s41598-018-35296-5

**Published:** 2018-11-19

**Authors:** Wadood Haq, Johannes Dietter, Eberhart Zrenner

**Affiliations:** Centre for Ophthalmology, Institute for Ophthalmic Research University of Tübingen, Elfriede-Aulhorn-Str. 5-7, D-72076 Tübingen, Germany

## Abstract

Electrical (e-) stimulation is explored in schemes to rescue the vision of blind people, e.g. those affected by Retinitis Pigmentosa (RP). We e-activated subretinally the surviving degenerated photoreceptors (d-Phrs) of the *rd1* mouse (RP model) and evoked visual responses in the blind retina. The e-stimulation was applied with a single platinum/iridium electrode. The d-Phrs (calcium-imaging) and ganglion cells (GC) activity (MEA-recording) were recorded in simultaneous multilayer recordings. The findings of this study confirm that the d-Phrs responded to e-stimulation and modulated the retinal network-activity. The application of blockers revealed that the synaptic interactions were dependent on voltage-gated calcium channels and mediated by the transmitters glutamate and GABA. Moreover, the gap junctions coupled networks promoted the lateral-spread of the e-evoked activity in the outer (~60 µm) and inner (~120 µm) retina. The activated GCs were identified as subtypes of the ON, OFF and ON-OFF classes. In conclusion, d-Phrs are the ideal interface partners for implants to elicit enhanced visual responses at higher temporal and spatial resolution. Furthermore, the retina’s intact circuity at the onset of complete blindness makes it a tempting target when considering the implantation of implants into young patients to provide a seamless transition from blinding to chip-aided vision.

## Introduction

Millions of people worldwide have lost their eyesight due to hereditary, blinding diseases. For example, in retinitis pigmentosa (RP), the retina’s light perceiving photoreceptor cells degenerate progressively, leading eventually to complete blindness. Basically three strategies are considered for restoring vision loss: (1) Gene therapy and optogenetics^1,2^, (2) cell transplantation^3–5^ and (3) e-implants with two types of retina interface: The subretinal implant^6,7^ and the epiretinal implant^8,9^. In this study, we focused on the approach of optimising subretinal e-stimulation. The subretinal implant has been beneficial for blind patients in clinical trials by providing visual sensations for daily-life activities^7^. Despite this pioneering success, the implant technology faces two different types of problems: (1) Poor temporal and spatial resolution, due to the retinal implant’s technical capability (e.g. 1500 electrodes each 50 × 50 µm^10^ and (2) the trial participants’ degree of retinal degeneration (e.g. blind for decades^7^). RP-induced retinal degeneration progresses over time with devastating retinal remodelling^11^. At the advanced stage of RP, the retinal circuity is compromised by morphological alterations^12,13^, e.g. loss of glutamate receptor sensitivity^14^ and strong sprouting of Müller glial cells^11^, which form an isolating layer at the neuronal–chip interface site. Hence, in our work, we investigated the degenerated, blind, retinal network’s functional capacity in order to estimate an optimal chip implantation phase during RP. In the *rd1* mouse model of human RP^15^, rod photoreceptor dystrophy (P10-20^16^) is accompanied by secondary cone photoreceptor degeneration. In the *rd1* mouse retina, light-evoked activity is absent at P20^17–19^, because the light-sensitive cone segments have degenerated. Nevertheless, a few layers of light-insensitive cone cell bodies with axon terminals^20^ (d-Phrs) survive in the blind retina for over one year^1,21^. Thus, we investigated a novel concept of interfacing the outer retina using e-implants at the onset of the RP-dependent, complete blindness, by employing single microelectrodes e-stimulating subretinally the blind retina of the *rd1* × HR2.1:TN-XL mouse model.

When e-stimulation was applied by a single platinum/iridium electrode, the *rd1* mouse’s fluorescent-labelled d-Phrs exhibited responses in calcium-imaging recordings. Simultaneously, MEA-recordings of the GCs were made to capture the e-stimulation mediated retinal signalling: The correlation of the “signal input” (e-stimulation of d-Phrs) and “signal output” (GC responses). The application of specific pharmaceutical agents revealed that the e-activated d-Phrs promoted network-mediated GC activity, which was facilitated by the vertical (1) and the horizontal transduction pathways (2) of the retinal signalling. (1) The vertical retina-signalling is synaptic (d-Phr => bipolar cell => GC) and relies on voltage-gated calcium channels. The inhibition of glutamate signalling prevented GC responses, while the inactivation of GABAergic inhibition led to elevated GC activity. (2) The horizontal spread of activity through the retina is mediated by gap-junction coupled cell-networks in the outer and inner retina. Finally, the activated GCs were identified as subtypes of the ON, OFF and ON-OFF cell classes.

Overall, the outcome of this study suggests that the blind *rd1* retinal network is still functional at the onset of complete blindness. Therefore, early implantation of a subretinal chip is recommended to provide a seamless vision aid. Furthermore, the low d-Phrs e-activation values permit the use of denser electrode fields, featuring smaller e-stimulation electrodes, and thereby improve the performance of the subretinal implants in terms of spatial and temporal resolution.

## Results

**Figure 1:**
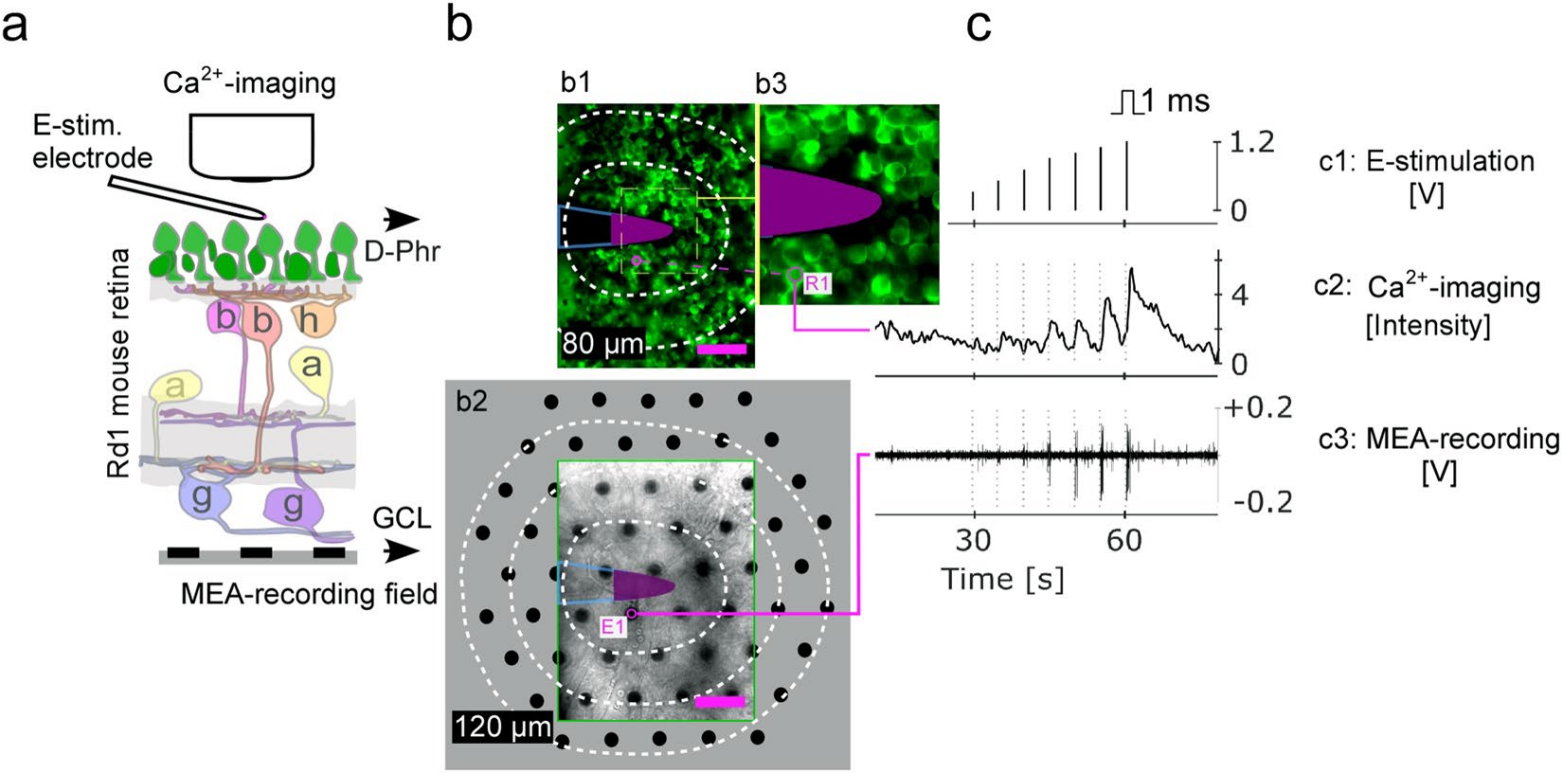
Subretinal e-stimulation of the *rd1* mouse retina and recordings. (**a**) Experimental layout: e-stimulation of the d-Phrs and simultaneous recording of the d-Phrs (calcium-imaging) and GCs (MEA-recordings). (**b**) The two recording planes: (b1) D-Phr layer: The stimulation electrode (tip: magenta) contacts the d-Phrs (green, TNXL labelled). (b2) The GC layer: Through the retina (green box corresponds to b1) the partial MEA-electrode-field is visible (black dots, grey: complete MEA-field). Projection of the e-stimulation electrode (b1) on the MEA-field. Both recording planes are divided into concentric fields (white dotted lines): The d-Phr plane is binned at 20 µm area (b1, 40 µm shown) and the MEA-field is binned at 40 µm area (b2). (b3) Zoom of the d-Phr layer nearby the stimulation electrode. (**c**) For each stimulation voltage-step (c1), a d-Phr (c2) and correlating GC response (c3) are shown. E-stimulation (c1): 0.3, 0.5, 0.7, 0.9, 1.0, 1.1 and 1.2 volts, anodic monophasic, 1 ms duration and 5 second interval. Abbreviation: D-Phrs: degenerated cone photoreceptors, b: bipolar cells, h: horizontal cell, a: amacrine cells and g, GC: ganglion cell; MEA: multi electrode array; e: electrical. Scale bar 40 µm. MEA-layout: 59 electrodes, 40 µm spacing distance and 10 µm diameter.

The d-Phrs of the *rd1* mouse retina in subretinal configuration were e-stimulated by a single platinum/iridium electrode, and both the e-activated d-Phrs’s calcium responses and the network-mediated correlated spike responses of the GCs were simultaneously recorded (Fig. 1). The compositions of the network mediated retinal signalling were identified as glutamatergic and GABAergic for vertical retina signalling while the gap junctional (GJ) cell coupling was responsible for the lateral retinal signalling in the outer and inner retina, which was revealed by antagonising each signalling pathway with respective pharmaceutical drugs.

### The d-Phrs are activated by e-stimulation

The *rd1* retina’s light-insensitive d-Phrs^17,18^ are responsive to e-stimulation, applied by a single electrode (Fig. 1a,b1). The d-Phrs were e-stimulated subretinally using an increasing voltage ramp of 0.3–1.2 V (Fig. 1c1 and c2) and two d-Phrs response parameters were evaluated: the number of responsive d-Phrs and their distance from the stimulation electrode. Control experiments were used to ensure that the recorded *rd1* retina-patch (P20) was blind and unresponsive to UV light illuminated during the calcium-imaging recordings, as shown previously for white-light stimulation^17,19^. The application of UV light flashes at 380 nm and 3 sec duration yielded no retinal responses.

**Figure 2:**
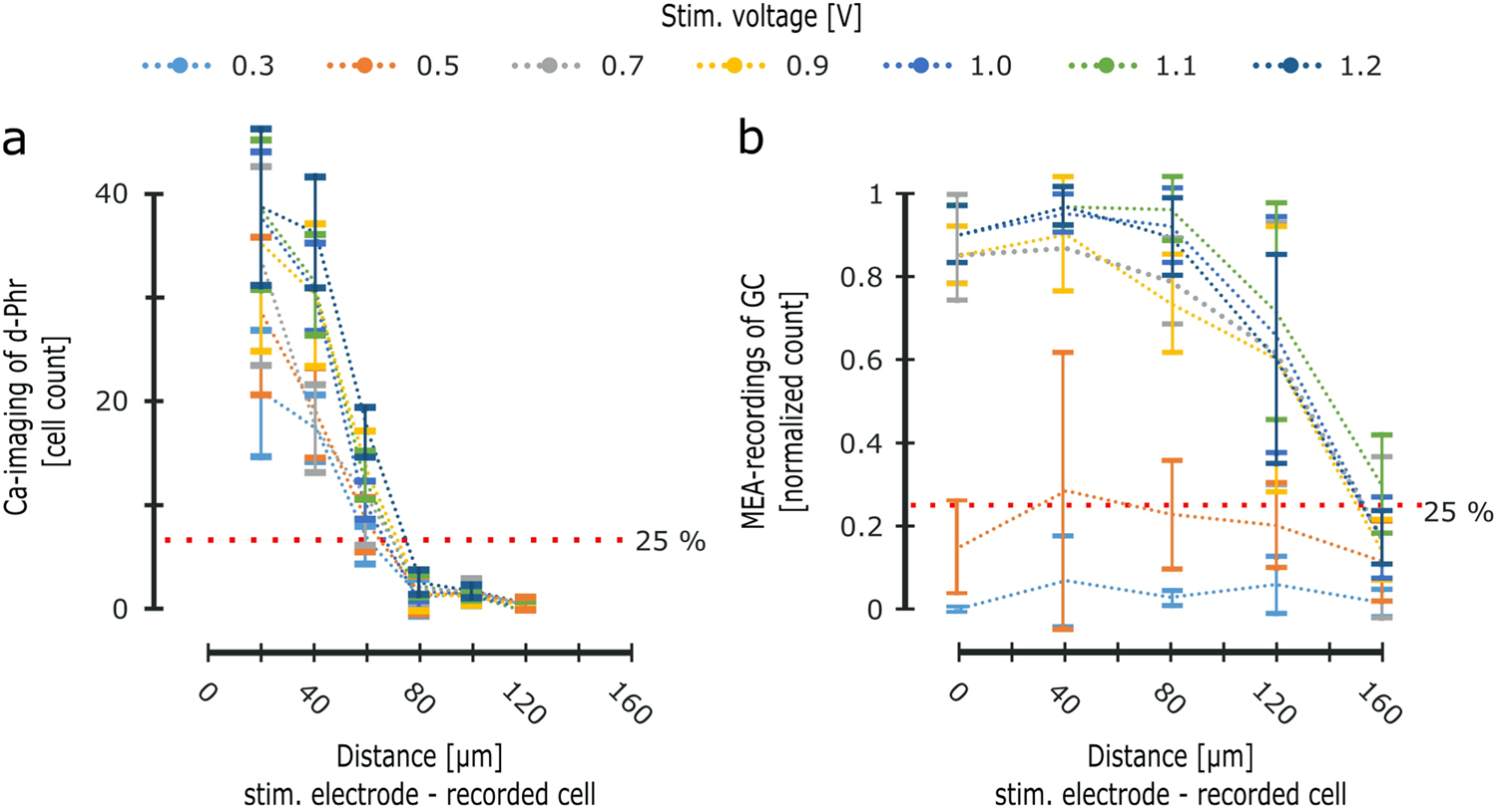
D-Phr and GC responses elicited by subretinal e-stimulation. Simultaneous Calcium-imaging recordings of *rd1* mouse d-Phrs (**a**) and MEA-recordings of GCs (**b)** during subretinal application of a voltage ramp (5 retina recordings, monophasic anodic pulse, 1 ms duration). (**a**) The ordinate represents the average number of e-activated d-Phrs within the respective binning field (see Fig. 1b1), whereas the abscissa represents the distance of e-activated d-Phrs from the stimulation electrode (20 µm binning, see Fig. 1b1). The red dotted line indicates a 25% threshold value (at 1 volt within 20 µm range). (**b**) The ordinate represents the average percentage of MEA-electrodes detecting e-stimulation dependent GC activity (normalised for each binning field of 40 µm, see Fig. 1b2). The abscissa represents the distance from the MEA-electrode to the stimulation electrode. Therefore, the bin “null” indicates MEA-electrodes, which are located directly under the stimulation electrode (see Fig. 1b2). The red dotted line indicates a 25% threshold value. Abbreviation: D-Phr: degenerated cone photoreceptors; GC: ganglion cell; MEA: multi electrode array; e-: electrical. Error bars indicate mean ± SEM. Tested by two-tailed Student’s t-test.

D-Phrs were responsive to e-stimulation at 0.3 V, and according to the incremental voltage step applied, the number of e-activated d-Phrs increased on average by 8.13 ± 5.25 cells (Fig. 2a). The increase in the number of e-activated d-Phrs from one voltage step to the next, was not significant (46.85 ± 14.32, 57.75 ± 17.92, 64.05 ± 21.40, 80.2 ± 23.97, 81.55 ± 24.23, 85.9 ± 24.46 and 95.6 ± 26.93).

The number of e-activated d-Phrs declined the spatial distance from the stimulation electrode increased, in a half-sigmoid curve (at all voltage steps, Fig. 2a). The highest number of d-Phrs are e-activated inside the first binning range of 20 µm, i.e. the closest distance to the stimulation electrode. With increasing distance, the number of e-activated d-Phrs drops by 1.32 ± 0.29-fold at 40 µm binning range and by 2.37 ± 0.41-fold at 60 µm binning range. The strongest cell response −95.50% of the total e-activated d-Phrs — occurred within the 60 µm binning range (44.90%, 35.45% and 15.15% cells at 20, 40 and 60 µm, respectively). The few remaining e-activated d-Phrs (4.5%) are distributed across the distant binning ranges of 80–100–120 µm, with strong standard deviations (1.65 ± 0.51, 1.4 ± 0.22 and 0.2 ± 0.13 cells), indicating that the exterior binning area is far enough beyond the influence of e-stimulation to cross the threshold. In conclusion, d-Phrs are responsive to e-stimulation of 0.3–1.2 V and the e-evoked activity extends spatially up to 60 µm from the surrounding boundary of the stimulation electrode.

### Ganglion cell responses elicited by means of subretinal e-activation of the d-Phrs

Correlated GC spike activity was detected in the MEA-recordings during e-activation of the d-Phrs, (Fig. 1b2,c3). The voltage value (0.3–1.2 voltage ramp) activating d-Phrs and promoting robust network mediated GC activity, was determined in the recording field at the shortest distance to (beneath) the stimulation electrode (Fig. 1b2). When the voltage ramp was applied, the first GC activity was detected at 0.5 V, on a few MEA-electrodes (15 ± 11.18%, Fig. 2b). At the increased stimulation voltage (0.7), significantly more MEA-electrodes detected GC activity (85 ± 6.8%). At higher stimulation voltages of 0.9–1.2 V, up to 90% of the MEA-electrodes detected GC activity. This data indicates that no significant increase in GC activity occurs between 0.7 to 1.2 V. Thus, the voltage value of 0.7 V was established as the minimum required for sufficient e-activation of the retinal d-Phrs, to induce network mediated GC activity. On the other hand, the number of e-activated GCs dropped with increasing distance from the stimulating electrode: Around 85% of the MEA-electrodes detecting GC activity were within the distances 80 µm (88 ± 6.8%, 93 ± 8.0% and 86 ± 9.6% at binning of 0, 40 and 80 µm, respectively; average over the 0.7–1.2 V steps). At a more distant binning of 120 µm, the number of MEA-electrodes detecting GC activity dropped to 63.7 ± 28.5% with larger standard deviation. At 160 µm, the number of MEA-electrodes detecting GC activity dropped significantly, with a high standard deviation (19.14 ± 10.83%), indicating that the exterior binning area is beyond sufficient e-stimulation influence. Thus, the distance study of this research suggests that the d-Phrs-induced network activity in the GC layer spreads up to 120 µm from the stimulation electrode.

### The e-evoked d-Phr responses and network activity rely on VGCC

**Figure 3:**
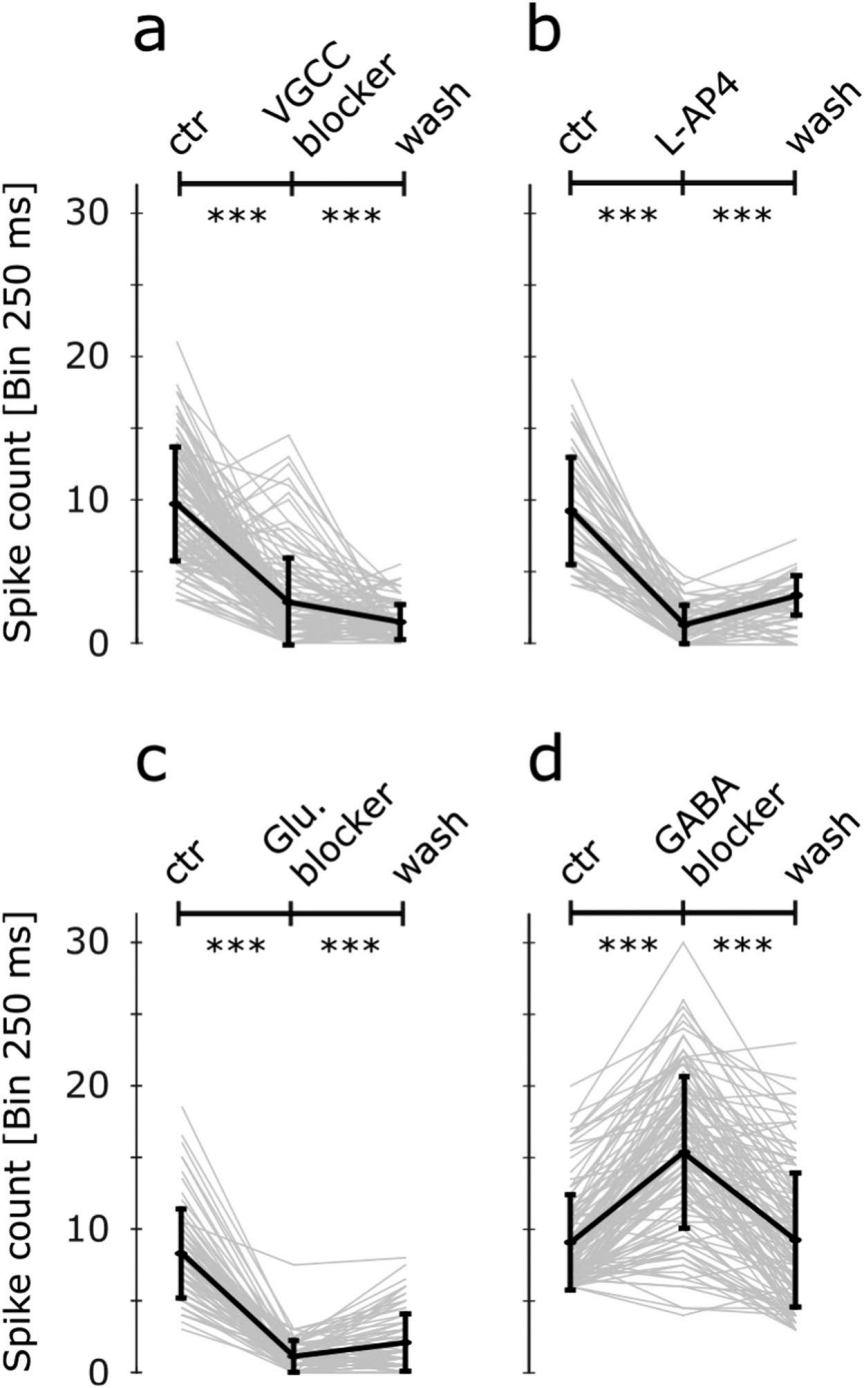
Drug-induced modulation of GC spike activity evoked by e-stimulation of d-Phrs. Three neuronal signalling mechanisms of the retinal vertical transduction pathways were investigated using respective pharmacological agents. The d-Phrs were e-stimulated subretinally and the post-stream GC activity was recorded by the MEA-system. The experiments were carried out for each drug before (control), after drug application (10 min) and wash (20 min) condition. The GC response data presented (spike counts, bin 250 ms) were collected at 1 Volt and 1 ms anodic monophasic stimulation. The drugs applied were (**a)** verapamil [100 µM] a VGCC antagonist (n = 101 GC, 4 retina recordings), (**b)** metabotropic glutamate receptor (mGluR6) agonist L-AP4 [100 µM] (n = 62 GC, 5 retina recordings), (**c)** glutamate blockers: mGluR6 agonist L-AP4 [100 µM], EAAT5 antagonist DL-TBOA [75 µM] and iGluR blocker NBQX [20 µM] (n = 70 GC, 4 retina recordings) and (**d)** GABAa/c receptor antagonist GABAzine [10 µM] and TPMPA [50 µM] (n = 116 GC, 5 retina recordings). D-Phr: degenerated cone photoreceptors; GC: ganglion cell; MEA: multi electrode array; e-: electrical; VGCC: voltage-gated calcium channel. Error bars indicate mean ± SEM. Significance: ***p ≤ 0.001. Tested by Wilcoxon signed-rank test.

So far, it has been established that the subretinal e-stimulation of the *rd1* retina evoked correlated responses in d-Phrs (calcium-imaging) and GC (MEA-recordings, Fig. 1), although with different spread range. To determine whether the GC activity is network mediated, the synaptic signal transmission was antagonised by the application of the voltage-gated calcium channel (VGCC) blocker verapamil^22,23^. The e-evoked calcium responses of the d-Phrs were reduced in the presence of verapamil, but the GC activity reduced significantly (Fig. 3a, average GC response spike activity at ctr: 10.14 ± 4.15, drug: 3.3 ± 2.41 (***) and wash: 2.05 ± 1.43 (***)). These results suggest that the correlated GC responses due to e-stimulation of d-Phrs, are network mediated and rely on the neuronal synapses VGCC.

### E-activated d-Phrs drive the postsynaptic bipolar cell activity by glutamate release

E-activated d-Phrs drive retinal network activity by releasing glutamate at the first synapse. The role of glutamate was investigated by applying various receptor agonist and antagonists and blocking the glutamatergic input of bipolar cells from d-Phrs. Identified ON bipolar cells (see section Network mediated activation of different ganglion cells types) were agonised by LAP-4 (mGluR6 agonist), leading to the suppression of ON-GC responses (Fig. 3b, average GC response spike activity at ctr: 9.35 ± 3.66, L-AP4: 1.35 ± 1.18 (***) and wash: 3.39 ± 1.63 (**)). The response-recovery was significant, but the initial response strength was not completely recovered (~40%). Next, the glutamate reception at the first synapse was completely prevented with a solution containing L-AP4, L-TBOA and NBQX. The ON bipolar cells were agonised by L-AP4 (mGluR6) and additionally antagonised by L-TBOA (EAAT5 receptors). In the *rd1* retina, the rod bipolar cells are contacted by d-Phrs via functional ectopic synapses, and promote excitatory rod BC (EAAT5 receptors) signalling by glutamate^18,24^. The OFF system was antagonised by NBQX (iGluR). The application of these drug combinations significantly reduced the GC post stream network dependent activity (Fig. 3c, average GC response spike activity at ctr: 8.39 ± 3.63, drug: 1.53 ± 1.89 (***) and wash: 0.93 ± 1.09 (**)). After the wash, no recovery of network mediated GC signals was observed, instead the blockage effect strengthened over time by further reduction of the GC activity.

### The signalling of d-Phrs to bipolar cells is modulated by GABAergic inhibition

At the first synapse, glutamate released by photoreceptors, regulates the function of the horizontal cells which in turn release the inhibitory transmitter, GABA, which hyperpolarises bipolar cells. Since the GABAergic inhibitory mechanism functions in the degenerated *rd1* outer retina^18^, e-stimulation experiments were performed in the presence of GABA receptor blockers (GABAc blocker TPMPA, and GABAa blocker Gabazine). In the absence of GABAergic inhibition of the bipolar cells, the GC activity response increased significantly upon e-stimulation of d-Phrs (Fig. 3d, average GC response spike activity at ctr: 9.07 ± 3.33, drug: 15.37 ± 5.29 (***) and wash: 9.21 ± 4.67 (***)). It was established that GABAergic suppression of bipolar cells is prominent in the *rd1* retina and effectively modulates the post stream network activity to the d-Phrs; through horizontal cells at the first synapse and presumably, through amacrine cells in the inner retina.

### Gap junctional connectivity contributes to the spatial spread of the e-evoked network-activity

**Figure 4:**
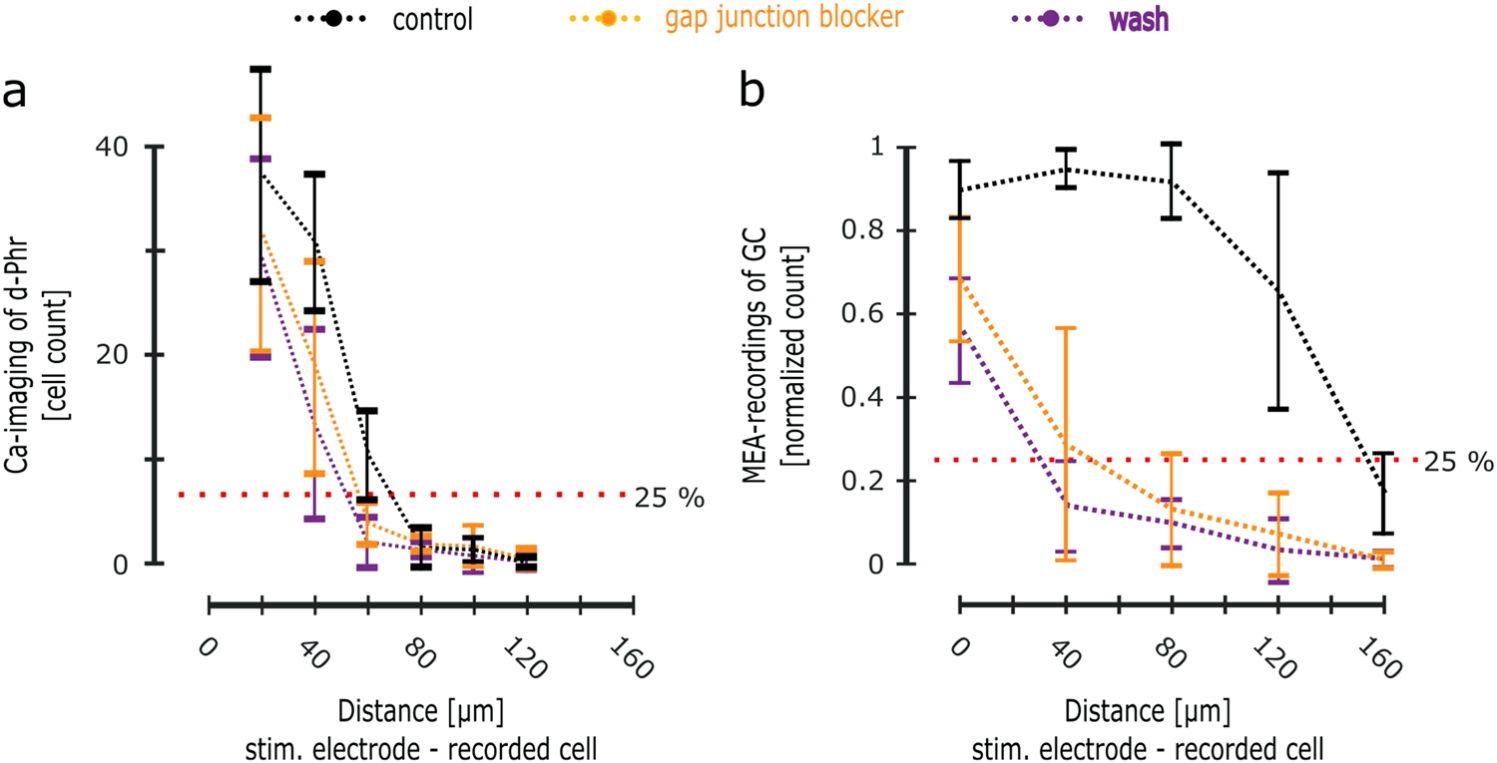
Modulation of the spatial spread of the e-evoked retinal network activity by gap junction blocker. Simultaneous calcium-imaging recordings of *rd1* mouse d-Phrs (**a)** and MEA-recordings of the GCs (**b)** for subretinal application of 1 Volt anodic monophasic pulse (1 ms duration) before (control), during the application of gap junction blocker carbenoxelone (50 µM, 10 min) and after wash (20 min) condition (5 retina recordings). (**a)** The ordinate represents the average number of e-activated d-Phrs, whereas the abscissa represents the distance between the e-activated d-Phrs and the stimulation electrode (20 µm binning, see Fig. 1b1). The red dotted line indicates a 25% threshold value (at 1 Volt within 20 µm range). (**b**) The ordinate represents the average percentage value of the MEA-electrodes detecting e-stimulation-dependent GC activity (normalised for each binning field, see Fig. 1b2). The abscissa represents the distance between the MEA-electrode and the stimulation electrode (40 µm binning). Therefore, the bin “null” indicates MEA-electrodes, which are situated directly under the stimulation electrode (see Fig. 1b2). The red dotted line indicates a 25% threshold value. Abbreviation: D-Phr: degenerated cone photoreceptors; GC: ganglion cell; MEA: multi electrode array; e-: electrical. Error bars indicate mean ± SEM. Tested by two-tailed Student’s t-test.

In a healthy retina, the horizontal spread of neuronal activity is facilitated by the electrical synapse, the GJs^25^. The *rd1* d-Phrs (Phr-Phr) and the horizontal cells (Hz-Hz) build GJ coupled networks in the outer retina^18^, while the amacrine cells (AII-AII) and GCs (GC-GC) create GJ coupled networks in the inner retina^25,26^. Thus, the role of the GJ in the lateral spread of the e-evoked network activity was examined pharmacologically by using carbenoxelone (CBX, gap junction connection blocker). Upon subretinal e-stimulation of the *rd1* retina, the following horizontal spread of cell activity from the stimulation electrode was observed: Up to 60 µm at the stimulation site (d-Phrs) (Fig. 2a) and up to 120 µm at the retinal output site (GC) (Fig. 2b). The analysis of cell-electrode distance revealed that the lateral spread of the activity in the *rd1* retina is reduced in the presence of the GJ blocker CBX and restricted to the vicinity of the stimulation electrode (Fig. 4). The number of e-activated cells closer to the stimulation electrode is only slightly affected by CBX: 1.3-fold decrease in the number of d-Phrs and 1.2-fold decrease in the number of GCs (outer retina Fig. 4a: ctr: 37.2 ± 10.12 cells and CBX: 31.6 ± 11.17 cells; inner retina Fig. 4b: ctr: 90 ± 6.84%, CBX: 68.75 ± 14.90%). In the outer retina (Fig. 4a), the activity spread in the d-Phr-network is restricted by the effect of the CBX to 40 µm (32.09%, 18.9 ± 10.11 cells), from 60 µm under control conditions (27.95%). At larger distances, above 60 µm, the percentage of the activated d-Phrs dropped steeply (average number of activated d-Phrs is 2.1). A similar damping effect on the horizontal spatial-spread of activity was observed in the GCs of the inner retina (Fig. 4b): Reduction of the 120 µm activity boundary to 40 µm in the presence of CBX (29.17%). At larger distances, binning up to 80 µm, the GC activity dropped steeply with a large standard deviation (13.54 ± 13.45, 7.62 ± 9.9 and 1.4 ± 3.9%, respectively), indicating that the exterior binning area was significantly (***) beyond the range of adequate e-stimulation influence. No GC activity recovery effect from the CBX dependent GJ network blockage was detected after the 20-minute wash period. The blockage even strengthened in the inner retina over time, narrowing the activity spread to the vicinity of the stimulation electrode, covering almost 65.9% of the overall activity. In the outer retina, the range of the activated d-Phrs remained at ~40 µm covering 96.2% of the e-activated d-Phrs. Thus, the data obtained in this study suggest that the GJ coupled networks facilitate the lateral spread of the e-evoked activity in the outer and inner retina.

### Network mediated activation of different ganglion cells types

**Figure 5:**
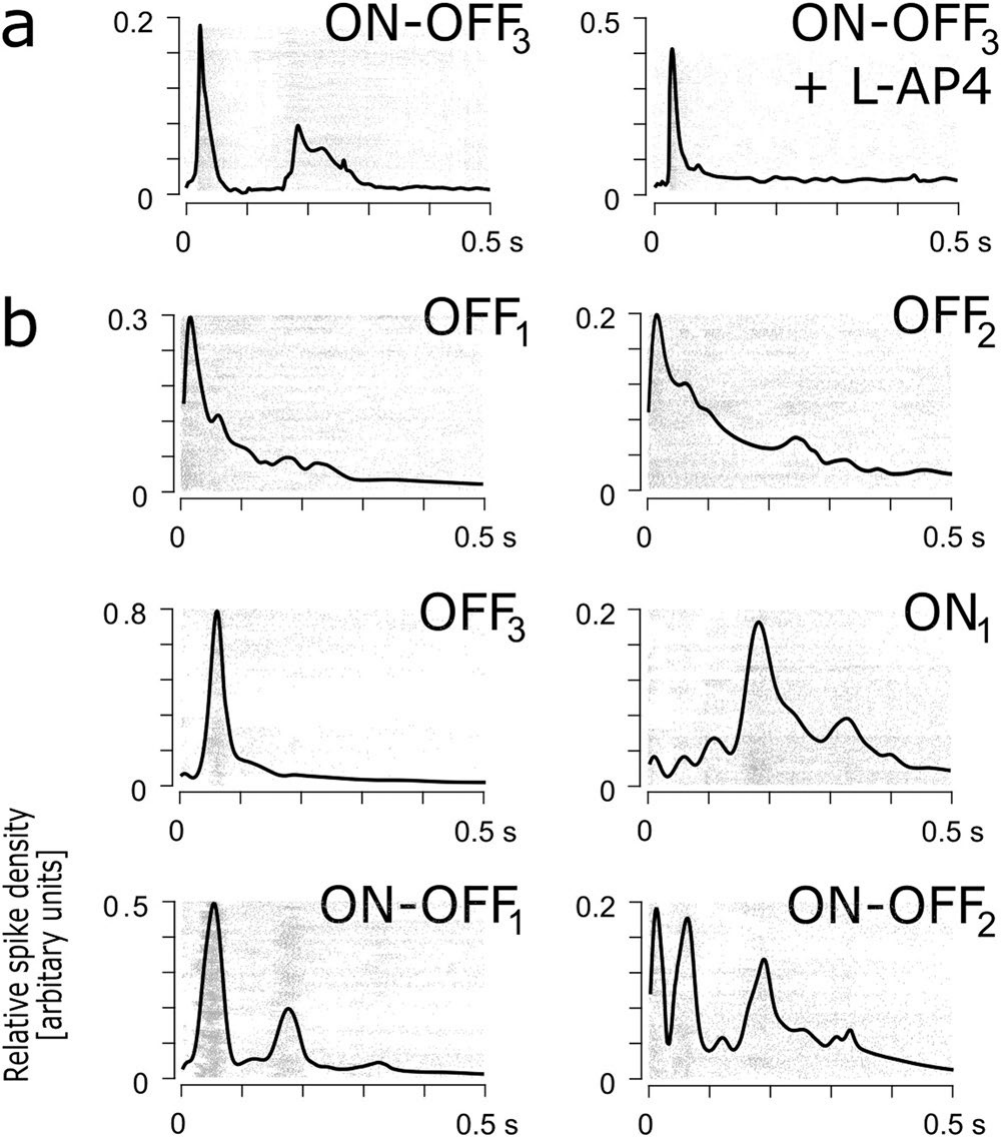
Different types of GCs activated by e-stimulation of d-Phrs. (**a**) Suppression of the ON-signal of the ON-OFF response-type by the mGluR6 agonist L-AP4 (n = 15 cells). (**b)** Clusters of six distinct types of *rd1* mouse GCs recorded at 0.7 Volt (monophasic anodic pulse, 1 ms duration) within 80 µm of the stimulation electrode (10 retina recordings). 30 repetitions of e-stimulation (5 sec period) were applied to identify each GC type by its spike density histogram. The numbers on the ordinate are relative to the maximum spike densities occurring in the spike trains. The solid graph line presents the density histogram of the GC cluster, and the spike raster plot presents the underlying cluster members. The GC subtypes type are classified as: OFF-sustain GCs (OFF_1_ and OFF_2_, n = 25; n = 13), OFF-transient GCs (OFF_3_, n = 18), ON-type GCs (ON_1_, n = 25) and ON-OFF types of GCs (ON-OFF_1_ and ON-OFF_2_, n = 27; n = 16). Y-axis is the arbitrary density unit. Abbreviation: D-Phr: degenerated cone photoreceptors; GC: ganglion cell; e-: electrical.

The basic GC types - ON, OFF and ON-OFF - were identified in the recordings of the subretinally e-stimulated blind retina (only GC within 80 µm distance from the e-stim electrode, stimulation voltage 0.7). In the healthy mouse retina, these three GC types are identified by their responses correlating to the state of the light stimulation (ON or OFF). However, for the GC type determination of the blind retina, we employed two different approaches. First, the cells were sorted by cluster analysis into two major types, with mono or biphasic response shape (Fig. 5). Second, the results from experiments with the ON-channel agonist were utilised to determine the functional types. For GCs with mono or biphasic response shape, it was observed, that in the presence of the ON-channel agonist L-AP4, only responses with larger latencies (Fig. 5a, peak at ~0.2 s) were suppressed, suggesting this signalling to be of the ON-type. Consequently, responses with shorter latency were assigned as OFF-type. Regarding the cells with the biphasic response shape, it is to note, even though the first response peak is of the OFF-type and the second of the ON-type, these cells are labelled as “ON-OFF” cells. Established this, the results of the classification analysis yielded six distinctive GC subtypes (Fig. 5b), four cluster types with mono-modality and two with multi-modality features. The cell type with the mono-modality feature were three OFF-types, including two OFF-sustained subtypes (OFF_1_ and OFF_2_) and one transient type (OFF_3_). The sustained sub-cell types OFF_1_ and OFF_2_ had response peaks at 16.7 and 20.9 ms after e-stimulation but differed in their sustained response durations of 93.7 and 155.6 ms (at 75% drop from response peaking time). With a small delay, the activity peak of the second cell OFF-type (OFF_3_, transient) was observed at 66.1 ms. The fourth cell cluster is of the ON-cell type (ON_1_) with an activity peak delay at 188.2 ms after application of the e-stimulus. The fifth and sixth cell type clusters were from the multi-modality feature group and represented two ON-OFF GC subtypes. The first ON-OFF_1_ cell type exhibited two peaks at 60.3 and 121.5 ms after e-stimulation, whereas the second type, ON-OFF_2_, featured three peaks: The first two close together at 18.7 and 49.7 ms and the third delayed at 125 ms after e-stimulation. Overall, the selective elimination of the ON-channel signalling (Figs 3b and 5a) suggests, that the GC activation is network mediated at the retina’s first synapse (d-Phrs => ON bipolar cells => GC). Therefore, the activation of different major GC types (ON, OFF and ON-OFF) proves that the network integrity of the RP-affected blind *rd1* mouse retina is preserved at a level which might provide enhanced visual sensations by means of e-activation of d-Phrs at the onset of RP-dependent, complete blindness.

## Discussion

In the *rd1* mouse model of human RP, rod-dystrophy leads to secondary cone degeneration^16^. During the initial disease phase, the photoreceptor cell layers degenerate, and only a few rows of segmentless cone cell bodies and their terminals survive in the blind retina^20,27^. In this study, the potential of these d-Phrs for restoring visual responses at the onset of complete blindness by means of subretinal e-stimulation, was examined.

The subretinal e-stimulation of *rd1* mouse d-Phrs by a single electrode elicited visual responses of the ON, OFF and ON-OFF type. Results of pharmacological blockers revealed that the e-stimulus correlated GC spike activity is network-mediated (d-Phr => BC => GC) by interneuronal synaptic interactions and not directly by e-stimulation. In addition, the activation of the OFF and subsequently the ON-channel demonstrates that the retinal network-activity originates from the d-Phrs: E-stim of the d-Phrs induced a depolarisation of the d-Phrs, followed by their subsequent hyperpolarisation. The dynamic of the relatively fast OFF-response caused by electrically induced depolarisation of d-Phrs is consistent with the relatively fast rise of the calcium influx (=>glutamate release). The long latency of the ON-signal (peak at ~0.2 s) is also consistent with the very slow recovery of the calcium levels (slow hyperpolarisation => less glutamate release). In turn, glutamate released by the d-Phrs drives the activity of the cells contacting into the outer plexiform layer: Horizontal cells (iGluR), ON (mGluR6) and OFF (iGluR), but also the rod bipolar cells (mGluR6 and EAAT5). It should be noted that the d-Phrs and rod bipolar cells of the *rd1* mouse established a miss-wired connection, which is sign-conserving and regulated by the glutamate transporter EAAT5^18^. In addition, the GABAergic inhibition modulates the synaptic input of the bipolar cells in the *rd1* outer retina^18^. In the presence of the GABA inhibitor, the subretinal e-activation of the d-Phrs leads to increased GC spike responses. This result agrees with previous findings, suggesting a vigorous, activity modulating, role of horizontal cells in the degenerated *rd1* retina^18^ and GABAergic inhibition in the inner retina of the P23H rat model of RP^28^. Overall, the present glutamatergic and GABAerig signalling as well as the basic visual responses of the ON, OFF and ON-OFF type (~40 mouse GC types^29^) are strong indications for an intact vertical signal transduction pathway of the blind mouse retina at the onset of the complete blindness.

Along the morphological changes, the emergence of spontaneous, light independent activity in the inner and outer *rd1* retina has also been reported^18,26^. The data obtained in this study do not support the expected negative interference from the auto–activity emerging from the *rd1* retina on the retinal activity evoked by subretinal e-stimulation of the d-Phrs. The spontaneous activity described is not stationary; rather it emerges locally in an epochal manner in “generator” cells and travels in a wave-like manner through the GJ coupled cells of the outer and inner retina^17,18,26^. Once the wave has disappeared^17^, the e-stimulation area becomes silent. Thus, we were able to e-activate the remaining degenerated retinal network in a noise-free condition and analyse the underlying mechanism of the functional circuity by the application of pharmacological agents. Furthermore, in this study’s calcium-imaging recordings, the responses in d-Phrs evoked by e-stimulation were much stronger than the calcium amplitudes generated by spontaneous activity in the same cell (Fig. 1c calcium trace). This finding establishes the dominance of e-evoked calcium responses over the spontaneous activity in the outer retina, since like the e-evoked activity of the d-Phrs, the spontaneous activity of the d-Phrs also relies on the VGCC^18^.

Furthermore, when subretinal e-stimulation of the d-Phrs is applied, the induced activity spreads horizontally from the stimulation electrode: In the outer (~60 µm) and inner (~120 µm) retina (ratio 1:2). This outcome suggests that the spread of activity around the stimulation electrode is based on horizontal cell signalling, conducted by GJ cell coupling. The GJ distance study data (control: Enlarged GJ coupling – CBX: Reduced GJ coupling) support the findings describing the GJ connectivity during visual adaptation (night: Enlarged GJ coupling – day: Reduced GJ coupling^25,30^). Due to the loss of light-sensitive photoreceptor segments, the *rd1* retina experiences the dark condition continuously, hence presumably the GJ coupling of the d-Phrs is adapted to the dark condition. The e-induced d-Phrs activity extends over a radius of 60 µm via the GJ coupled d-Phr-network, which is a 60% match to the GJ-coupled connectivity range of the photoreceptors (100 µm) in healthy mouse and rabbit retina in darkness^31^. Similarly, increased GJ connectivity during light adaptation, has been previously reported for turtle cone-cone coupling^32^, and rod-cone coupling in salamanders^33^. In contrast, the photoreceptor coupling is restricted to few neighbouring cells in the presence of light, corresponding to the CBX effect observed in this study’s experiments: CBX restricts the lateral spread of the e-evoked activity in the GJ coupled network of d-Phrs to few cells. Moreover, the cone-driven horizontal cell coupling is modulated during the light adaption processes^34–36^. In the *rd1* retina, the GJ coupled networks of the horizontal cells and d-Phrs, are capable of signalling over a larger distance and performing synchronised cell activity^18,37^. Hence, it is conceivable that the horizontal cells also contribute to the lateral spread of the e-evoked activity in the outer retina. In the inner retina, the e-evoked activity in the GC layer extends up to 120 µm from the stimulation electrode. It is very likely that in the *rd1* retina, both bipolar cell pathways (d-Phrs => rod & cone bipolar cell^18,38^) feed the e-evoked activity of d-Phrs into the inner retina’s two GJ coupled networks: The amacrine cell network and GC network^25^. These two networks are known to generate synchronised spike activity in neighbouring retinal GCs^39^, and therefore it is very likely that they are responsible for the twice as large activity spread in the inner retina in comparison to the outer retina. After the 20-minute CBX wash, the GJ blockage did not diminish but became stronger. A similar outcome had previously been observed in *rd1* retina recordings^18^ and discussed as a retinal degeneration-effect leading to increased cell vulnerability. It is important to note that a further effect of CBX on VGCC has also been reported, affecting the cone and horizontal cell signalling^37^. We took this potential side effect into account in the data evaluation, by comparing the GJ blocker data (CBX) and VGCC blocker data (Verapamil): CBX evidently affected the lateral spread of the activity by restricting it to the vicinity of the stimulation electrode, whereas verapamil completely blocked the synaptic transmission, silencing the network-mediated GC-responses. A second GJ blocker, meclofenamic acid (MFA) has previously been reported to eliminate spontaneous activity in *rd10* mouse recordings and preserve retinal light responses^40^. However, in this study’s experiments, MFA had a very strong impact on the *rd1* retina, silencing the retinal activity in calcium-imaging and MEA-recordings. It is conceivable that the activity-diminishing effect of MFA on the *rd1* retina recordings, is due to the degeneration dependent vulnerability of the *rd1* retinal circuity, when compared to *rd10* recordings at the same age.

Overall, the e-activation of d-Phrs is of great relevance for blind RP patients. In the initial phase of RP, the d-Phrs in *rd1* mouse^20^ and human retinas sprout^41^, even arranging a functional ectopic synapse between cones and rod bipolar cells^1,18,24^, indicating that these connections represent an effort by the bipolar cells to renew input activity. To restore vision by d-Phrs, they were resensitised to light in an optogenetic approach using channelrhodopsin^1^. The operational range of channelrhodopsin is at unnaturally high levels of light intensity and therefore not yet useful for human therapy without complex and extensive light amplification devices. The method employed in this study, e-activation of the d-Phrs using low and safe e-stimulation values, was successful in restoring network-mediated visual responses in different types of GCs. Thus, d-Phrs are a promising target for e-stimulation to substitute retinal afferent input in human therapy using established subretinal e-implants. Along the same lines, some patients have reported experiencing an outstanding visual perception with the e-chip implanted at the foveal area, e.g. recognising letters^6,10^ or facial expressions^42^. Thus, it is conceivable that the outstanding e-chip performance was due to the presence of the d-Phr in the foveal region providing intact glutamate transmission to the outer plexiform layer. In terms of the sustainability of therapeutic effects, the genetically introduced channel rhodopsin could not stop RP, so that the benefit is lost with progressing retinal degeneration^1^ while vision provided from subretinal implants^43^ with a technically overhauled implant Alpha AMS, remains stable over a long period. Additionally, e-stimulation has been reported to be beneficial in the treatment of human RP^44,45^ and neuroprotective in RP rat models^46^. Therefore, implantation of the subretinal implant at an early stage of RP-induced blindness while the d-Phrs are present is suggested, to deliver afferent input and neuroprotection at the same time and provide a seamless transition from natural seeing towards e-chip aided vision with a long-term perspective. Moreover, the lower e-stimulation values for d-Phrs activation allow smaller electrodes and therefore denser electrode arrays, enhancing the implants’ spatial resolution.

## Materials and Methods

### Animals

In this study, the crossbred mouse line HR2.1:TN-XL × rd1 (rd1^TN-XL^)^18,47^ homozygous for the *rd1* allele^15^ was used, to identify the fluorescently labelled degenerated cones (d-Phrs). The mouse line is referred to as “*rd1*”. The animals were housed under standard, white, cyclic lighting, had free access to food and water and were used irrespective of gender. Animal protocols, compliant with §4 of the German law on animal protection, were reviewed and approved by the Tübingen University committee on animal protection (Einrichtung für Tierschutz, Tierärztlichen Dienst und Labortierkunde; Registration No.: 21/01/2015). All the experiments were performed in accordance with the ARVO statement for the use of animals in ophthalmic and visual research. The animals were sacrificed in a carbon dioxide atmosphere following cervical dislocation. Experiments were performed at postnatal days (P) 20–50 (n = 17).

### Tissue preparation

The eyes of the *rd1* mice were enucleated and the retina was isolated in an extracellular solution containing (in mM): 125 NaCl, 26 NaHCO_3_, 2.5 KCl, 2 CaCl_2_, 1 MgCl_2_, 1.25 NaH_2_PO_4_, and 20 glucose, and maintained at pH 7.4 using carboxygen perfusion (95% CO_2_/5% O_2_). All the chemicals were obtained from Sigma-Aldrich (Germany). Retinal degeneration in *rd1* mice progresses with age and retinal eccentricity, therefore the recording fields were consistently chosen within an ~800 µm radius from the optic disc. The retinas were incubated in an extracellular solution containing 0.27 µM Fura-2-AM and 0.1% Pluronic acid (Invitrogen, Eugene, USA) for 35 minutes at room temperature, washed and transferred to the MEA-recording chamber (GC side down), where the tissue was perfused with carboxygenated medium at 32 °C. The chamber perfusion rate was adjusted to 1 ml/min.

### E-stimulation and recordings

In addition to subretinal e-stimulation, multilayer recordings in a “sandwich” configuration were performed simultaneously, by applying calcium-imaging and MEA recordings (Fig. 1a). See supplementary information for details. All data generated or analysed during this study are included in this published article (and its Supplementary Information files).

### Data analyses

Custom developed scripts (MATLAB, The MathWorks, Germany) were used for the data analysis of calcium-imaging, MEA-recordings and spatial activity spread, unless otherwise indicated. For GC-type analysis online available tools^48–53^ were modified and utilized. See supplementary information for details. The Wilcoxon signed-rank test and the two-tailed Student’s t-test were used for the statistical evaluation of drug effects; statistical significance is indicated as * for p ≤ 0.05, ** for p ≤ 0.01 and *** for p ≤ 0.001; and all parameters are given as mean ± SEM.

### Pharmacology

Pharmaceutical agents were applied via the perfusion system, in the recording chamber for 10 minutes and washed for 20 minutes. We used (in µM): 100 L-AP4 (mGluR6 agonist; L-2-amino-4-phosphonobutyric acid) and 20 NBQX (AMPA/kainate-type GluR antagonist; 2,3-Dioxo-6-nitro-1,2,3,4-tetrahydro-benzo[f]quinoxaline-7-sulfonamide) obtained from Bio Trend, 75 DL-TBOA (glutamate transporter antagonist DL-threo-β-Benzyloxy aspartic acid); 100 Verapamil (L-type voltage-gated calcium channel blocker), 50 TPMPA (GABA_C_ receptor antagonist; 1,2,5,6-Tetrahydropyridin-4-yl)met hylphosphinic acid) and 10 Gabazine (GABA_A_ receptor antagonist; 6-Imino-3-(4-methoxyphenyl)-1(6 H)-pyridazine-butanoic acid hydrobromide) obtained from TocrisBioscience, and 50 carbenoxolone (CBX, gap junction blocker; (3β,20β)-3-(3-Carboxy-1-oxopropoxy)-11-oxoolean-12-en-29-oic acid disodium) obtained from Sigma-Aldrich.

## Electronic supplementary material

Supplementary Information

## References

[1] Busskamp V (2010). Genetic Reactivation of Cone Photoreceptors Restores Visual Responses in Retinitis Pigmentosa. Science.

[2] Sahel JA, Roska B (2013). Gene therapy for blindness. Annu Rev Neurosci.

[3] Mandai M (2017). iPSC-Derived Retina Transplants Improve Vision in rd1 End-Stage Retinal-Degeneration Mice. Stem Cell Reports.

[4] Jayakody SA, Gonzalez-Cordero A, Ali RR, Pearson RA (2015). Cellular strategies for retinal repair by photoreceptor replacement. Prog Retin Eye Res.

[5] Jeon S, Oh IH (2015). Regeneration of the retina: toward stem cell therapy for degenerative retinal diseases. BMB Rep.

[6] Zrenner E (2011). Subretinal electronic chips allow blind patients to read letters and combine them to words. P Roy Soc B-Biol Sci.

[7] Stingl K (2015). Subretinal Visual Implant Alpha IMS–Clinical trial interim report. Vision research.

[8] da Cruz L (2013). TheArgus II epiretinal prosthesis system allows letter and word reading and long-term function in patients with profound vision loss. The British journal of ophthalmology.

[9] Humayun MS (2012). Interim results from the international trial of Second Sight’s visual prosthesis. Ophthalmology.

[10] Zrenner E (2013). Fighting blindness with microelectronics. Science translational medicine.

[11] Marc RE, Jones BW, Watt CB, Strettoi E (2003). Neural remodeling in retinal degeneration. Prog Retin Eye Res.

[12] Strettoi E, Porciatti V, Falsini B, Pignatelli V, Rossi C (2002). Morphological and functional abnormalities in the inner retina of the rd/rd mouse. Journal of Neuroscience.

[13] Cuenca N, Pinilla I, Sauve Y, Lund R (2005). Early changes in synaptic connectivity following progressive photoreceptor degeneration in RCS rats. European Journal of Neuroscience.

[14] Strettoi E, Pignatelli V, Rossi C, Porciatti V, Falsini B (2003). Remodeling of second-order neurons in the retina of rd/rd mutant mice. Vision research.

[15] Bowes C (1990). Retinal Degeneration in the Rd Mouse Is Caused by a Defect in the Beta-Subunit of Rod Cgmp-Phosphodiesterase. Nature.

[16] Carter-Dawson LD, LaVail MM, Sidman RL (1978). Differential effect of the rd mutation on rods and cones in the mouse retina. Investigative ophthalmology & visual science.

[17] Stasheff SF (2008). Emergence of sustained spontaneous hyperactivity and temporary preservation of OFF responses in ganglion cells of the retinal degeneration (rd1) mouse. Journal of neurophysiology.

[18] Haq W, Arango-Gonzalez B, Zrenner E, Euler T, Schubert T (2014). Synaptic remodeling generates synchronous oscillations in the degenerated outer mouse retina. Frontiers in neural circuits.

[19] Haq W, Dietter J, Bolz S, Zrenner E (2018). Feasibility study for a glutamate driven subretinal prosthesis: local subretinal application of glutamate on blind retina evoke network-mediated responses in different types of ganglion cells. Journal of neural engineering.

[20] Lin B, Masland RH, Strettoi E (2009). Remodeling of cone photoreceptor cells after rod degeneration in rd mice. Experimental eye research.

[21] Garcia-Fernandez JM, Jimenez AJ, Foster RG (1995). The persistence of cone photoreceptors within the dorsal retina of aged retinally degenerate mice (rd/rd): implications for circadian organization. Neuroscience letters.

[22] Ball SL, McEnery MW, Yunker AM, Shin HS, Gregg RG (2011). Distribution of voltage gated calcium channel beta subunits in the mouse retina. Brain research.

[23] Simms BA, Zamponi GW (2014). Neuronal Voltage-Gated Calcium Channels: Structure, Function, and Dysfunction. Neuron.

[24] Peng YW, Hao Y, Petters RM, Wong F (2000). Ectopic synaptogenesis in the mammalian retina caused by rod photoreceptor-specific mutations. Nature neuroscience.

[25] Bloomfield SA, Volgyi B (2009). The diverse functional roles and regulation of neuronal gap junctions in the retina. Nature reviews. Neuroscience.

[26] Trenholm S (2012). Intrinsic oscillatory activity arising within the electrically coupled AII amacrine-ON cone bipolar cell network is driven by voltage-gated Na+ channels. The Journal of physiology.

[27] Sanyal S, De Ruiter A, Hawkins RK (1980). Development and degeneration of retina in rds mutant mice: light microscopy. Journal of Comparative Neurology.

[28] Jensen RJ (2012). Blocking GABA(C) receptors increases light responsiveness of retinal ganglion cells in a rat model of retinitis pigmentosa. Experimental eye research.

[29] Baden T (2016). The functional diversity of retinal ganglion cells in the mouse. Nature.

[30] Smith RG, Freed MA, Sterling P (1986). Microcircuitry of the dark-adapted cat retina: functional architecture of the rod-cone network. The Journal of neuroscience: the official journal of the Society for Neuroscience.

[31] Ribelayga C, Cao Y, Mangel SC (2008). The circadian clock in the retina controls rod-cone coupling. Neuron.

[32] Copenhagen DR, Green DG (1987). Spatial Spread of Adaptation within the Cone Network of Turtle Retina. J Physiol-London.

[33] Yang XL, Wu SM (1989). Modulation of rod-cone coupling by light. Science.

[34] Baldridge WH, Weiler R, Dowling JE (1995). Dark-suppression and light-sensitization of horizontal cell responses in the hybrid bass retina. Visual neuroscience.

[35] Lankheet MJ, Przybyszewski AW, van de Grind WA (1993). The lateral spread of light adaptation in cat horizontal cell responses. Vision research.

[36] Bloomfield SA, Xin DY, Persky SE (1995). A Comparison of Receptive-Field and Tracer Coupling Size of Horizontal Cells in the Rabbit Retina. Visual neuroscience.

[37] Vessey JP (2004). Carbenoxolone inhibition of voltage-gated Ca channels and synaptic transmission in the retina. Journal of neurophysiology.

[38] Volgyi B (2013). Gap junctions are essential for generating the correlated spike activity of neighboring retinal ganglion cells. PloS one.

[39] Toychiev AH, Ivanova E, Yee CW, Sagdullaev BT (2013). Block of gap junctions eliminates aberrant activity and restores light responses during retinal degeneration. The Journal of neuroscience: the official journal of the Society for Neuroscience.

[40] Fariss RN, Li ZY, Milam AH (2000). Abnormalities in rod photoreceptors, amacrine cells, and horizontal cells in human retinas with retinitis pigmentosa. American journal of ophthalmology.

[41] Stingl K (2013). Artificial vision with wirelessly powered subretinal electronic implant alpha-IMS. Proceedings. Biological sciences/The Royal Society.

[42] Stingl K (2017). Interim Results of a Multicenter Trial with the New Electronic Subretinal Implant Alpha AMS in 15 Patients Blind from Inherited Retinal Degenerations. Frontiers in neuroscience.

[43] Robles-Camarillo D, Niño-de-Rivera L, López-Miranda J, Gil-Carrasco F, Quiroz-Mercado H (2013). The effect of transcorneal electrical stimulation in visual acuity: Retinitis pigmentosa. Journal of Biomedical Science and Engineering.

[44] Schatz A (2011). Transcorneal electrical stimulation for patients with retinitis pigmentosa: a prospective, randomized, sham-controlled exploratory study. Investigative ophthalmology & visual science.

[45] Morimoto T (2007). Transcorneal electrical stimulation promotes the survival of photoreceptors and preserves retinal function in royal college of surgeons rats. Investigative ophthalmology & visual science.

[46] Wei T (2012). Light-driven calcium signals in mouse cone photoreceptors. Journal of Neuroscience.

[47] Chua J, Fletcher EL, Kalloniatis M (2009). Functional Remodeling of Glutamate Receptors by Inner Retinal Neurons Occurs From an Early Stage of Retinal Degeneration. Journal of Comparative Neurology.

[48] Inayat S, Rountree CM, Troy JB, Saggere L (2015). Chemical stimulation of rat retinal neurons: feasibility of an epiretinal neurotransmitter-based prosthesis. Journal of neural engineering.

[49] Fukuda Y, Stone J (1974). Retinal distribution and central projections of Y-, X-, and W-cells of the cat’s retina. Journal of neurophysiology.

[50] Grün, S. & Rotter, S. *Analysis of parallel spike trains* (Springer, 2010).

[51] Lewicki MS (1998). A review of methods for spike sorting: the detection and classification of neural action potentials. Network.

[52] Carcieri SM, Jacobs AL, Nirenberg S (2003). Classification of retinal ganglion cells: a statistical approach. Journal of neurophysiology.

[53] Shimazaki H, Shinomoto S (2010). Kernel bandwidth optimization in spike rate estimation. J Comput Neurosci.

