# Supplementary material for: Electrical activation of degenerated photoreceptors in blind mouse retina elicited network-mediated responses in different types of ganglion cells
Source: Sci Rep. 2018 Nov 19;8:16998. doi: 10.1038/s41598-018-35296-5 (PMC6243018; doi:10.1038/s41598-018-35296-5)
Supplement: Supplementary file 1 — Supplementary Information [file 41598_2018_35296_MOESM1_ESM.pdf]

Title: Electrical activation of degenerated dormant-photoreceptors elicited network-mediated responses in different types of ganglion cells.

Wadood Haq*, Johannes Dietter, Eberhart Zrenner

E-stimulation and recordings. In addition to (i) subretinal e-stimulation, multilayer recordings in "sandwich" configuration was simultaneously performed, applying (ii) calcium-imaging and (iii) MEA recordings (Fig. 1a). i) E-stimulation: At the recording time of P20 – P50, the segments of the *rd1* mouse cones were degenerated (segmentless), and therefore the fluorescent TN-XL labelled cone somata (d-Phrs) are visible and methodically accessible from the distal whole mount surface (Fig. 1a and b1). Under microscopic vision control (40X), the e-stimulation electrode was positioned by a micromanipulator (5171, Eppendorf, Germany) to the outer retina in the recording chamber, electrode tip contacting the d-Phrs gently. Single metal electrodes were used to apply e-stimulation in subretinal configuration (Platinium/Iridium Macro Electrodes, tip ~ 3 µm, MicroProbes). Only stimulation electrodes of ~ 7 kOhms were used (NanoZ, Multi Channel Systems (MCS)). The e-stimulation protocol, a voltage ramp (0.3, 0.5, 0.7, 0.9, 1.0, 1.1 and 1.2 V) of single anodal monophasic pulses (5 seconds interval) was generated by the STG 2008 device using MC Stimulus II software (v 3.4.4, MCS). In experiments for the GC subtype determination, 30 repetitions of 0.7 V pulses were applied (5 seconds interval). ii) Calcium-imaging: Ratiometric calcium-imaging recordings were performed utilizing an upright fluorescence microscope (BX50WI, Olympus, Germany) equipped with a 40X water immersion objective (LUMPlan FL, 40X/0.80W, ∞/0, Olympus), a polychromator (VisiChrome, Till Photonics) and a CCD camera (RETIGA-R1, 1360×1024 pixel, 16 bit). During the calcium-imaging recording, stacks (single-plane two-channel) of the Fura-2 fluorescence in the outer retina were acquired at 10 Hz (λ_{exc}= 340 and 380 nm; Olympus U-MNU filter set, 30 ms exposure time, 8-pixel binning) using the VisiView software (Till Photonics). iii) MEA-recordings: GC activity was recorded via the USB-MEA60-Up-BC-System-E (MCS) equipped with HexaMEA 40/10iR-ITO-pr at 25000 Hz raw data sampling. The trigger synchronized operation of the e-stimulation, calcium-imaging and MEA-recording were controlled by the recording protocol set within the MCRack software (v 4.6.2, MCS) and the digital I/O – box (MCS): 60 sec only MEA-recording, followed by 60 sec MEA + calcium-imaging recording, then application of the e-stimulation pulses (+ trigger detection) and finally a 60 sec of post stimulus recording. Immediately after the recordings, two images of the recorded region were taken: The first image was recorded at the focal plane of the photoreceptor layer to visualize TN-XL-expressing d-Phrs (U-MSWG Olympus filter set) and the position of the stimulation electrode (Fig. 1b), while the second image was recorded at the deeper focal plane of the retinal GC layer to visualize the recording MEA-electrodes.

Data analyses: Calcium-imaging. Calcium-imaging ratio-stacks were generated by dividing the fluorescence images recorded at the excitation wavelengths of F_{340} and F_{380}. To detect the e-elicited calcium responses in the d-Phrs, fluorescent-labelled d-Phrs in the TN-XL image were manually encircled by regions of interest (ROIs) and the obtained ROIs coordinates were used to extract corresponding calcium traces from the ratio-stacks. The e-stimulation dependent d-Phrs calcium responses were detected by thresholding the post stimulus response (+300 ms) by a factor of 1.5 to pre stimulus activity (-1000 ms). Spontaneously active d-Phrs were detected, indicating a signal-to-noise ratio ≥ 10 (with the transient amplitude as signal and 1 SD of the baseline fluctuations as "noise"), during the control recording time prior to the start of the e-stimulation protocol [18].

Data analyses: MEA-recordings. MEA-recording files were high pass filtered at 200 Hz Butterworth 2nd order, and exported to *.hdf files by MC DataManager (v 1.6.1.0) for further data processing in MATLAB. A static threshold value of -20 µV was applied for spike detection, and only somal spikes with biphasic shape were selected [48,49]. Recording channels posing a median noise level larger than ± 10 µV were excluded from evaluation. Spike sorting for each MEA-electrode channel was performed to identify and isolate individual GCs [50]: Feature extraction via principal component analysis (PCA, MATLAB) and spike sorting by application of Gaussian clustering (MATLAB, [51]). The cell clustering results were verified by manual inspection. GC with aberrant spontaneous activity were

detected by calculating the pre-stimulus histogram (binning 50 ms) in the control recording phase prior to the start of the e-stimulation protocol – on average higher activity than the noise free channels. E-stimulation dependent GC activity was detected by thresholding the post stimulus spike histogram (+500 ms, average of 5 bins of 100 ms) by a factor of 1.25 to the pre-stimulus spike histogram (-1000 ms, average of 10 bins of 100 ms). To determine GC subtypes a self-developed method was applied. Generally, to identify GC of ON, ON/OFF and OFF types in healthy mouse retina, light flashes were applied in time dimension of seconds [52]. In the present study, the d-Phrs of the blind retina were e-activated by stimulation pulses of 1 ms duration. For GC type identification, 30 trails of e-stimulus presentations of each GC were averaged and peristimulus time histograms (PSTHs) were generated using the online available MATLAB code of [53]. Next, inspired by the GC classification approach of Carcieri [52], GC were sorted in uni-, bi-, and multi-modality classes using the convex hull of the PSTHs. Then GCs of each modality class were sub-clustered (MATLAB, gaussian clustering algorithm [51]). The final GC subtype cluster results were manually reviewed and approved.

Data analyses: spatial activity spread. To estimate the horizontal spatial spread of the e-evoked activity, the recording planes were divided into equal large concentric fields from the boundary of the stimulation electrode (Fig. 1b, electrode boundary hand marked and imported in MATLAB). The calcium-imaging field (recording d-Phrs) was binned at 20 µm areas (Fig. 1b1, representative 40 µm binning shown), whereas the MEA-electrode field (recording GCs) was binned at 40 µm areas (Fig. 1b2). Next, the distance of each d-Phrs utilizing ROI coordinates to the e-stimulation electrode boundary was calculated and the cell was counted to the respective distance category (Fig. 1). Analogously, the distance of the MEA-electrodes to the stimulation electrode was estimated (Fig. 1), utilizing the ROI coordinates of the MEA-electrodes. It is important to note that the calcium-imaging provides images at cellular resolution (Fig. 1b1). While, the MEA-recording field has a 40 µm electrode spacing resolution (Fig. 1b2), and therefore cell activity in between the electrodes is not detectable. In the same line, d-Phrs covered by the e-stimulation electrode are not recorded by calcium-imaging (Fig. 1b1), but the downstream activity of GC is detected by MEA-electrodes under the stimulation electrode. Thus the binning of "0" distance was introduced, indicating the MEA-electrodes overlapped by the reflection of the stimulation electrode (Fig. 1b2). The number of MEA-electrodes detecting GC activity within a bin (0, 40, 80, 120 and 160 µm) are normalized to the total number of electrodes located within the respective binning field (4, 12, 15, 14 and 14 electrodes).
